# Appoptosin Mediates Lesions Induced by Oxidative Stress Through the JNK-FoxO1 Pathway

**DOI:** 10.3389/fnagi.2019.00243

**Published:** 2019-09-04

**Authors:** Cuilin Zhang, Zhenqiu Tan, Yongzhuang Xie, Yingjun Zhao, Timothy Y. Huang, Zhaoping Lu, Hong Luo, Dan Can, Huaxi Xu, Yun-wu Zhang, Xian Zhang

**Affiliations:** ^1^Fujian Provincial Key Laboratory of Neurodegenerative Disease and Aging Research, School of Pharmaceutical Sciences, School of Medicine, Institute of Neuroscience, Xiamen University, Xiamen, China; ^2^The United Innovation of Mengchao Hepatobiliary Technology Key Laboratory of Fujian Province, Mengchao Hepatobiliary Hospital of Fujian Medical University, Fuzhou, China; ^3^Neuroscience Initiative, Sanford Burnham Prebys Medical Discovery Institute, La Jolla, CA, United States; ^4^Fujian Provincial Maternity and Children's Hospital of Fujian Medical University, Fuzhou, China

**Keywords:** appoptosin, oxidative stress, ROS, JNK, FoxO1

## Abstract

Oxidative stress is a common feature of neurodegenerative diseases and plays an important role in disease progression. Appoptosin is a pro-apoptotic protein that contributes to the pathogenesis of neurodegenerative diseases such as Alzheimer's disease and progressive supranuclear palsy. However, whether appoptosin mediates oxidative stress-induced neurotoxicity has yet to be determined. Here, we observe that appoptosin protein levels are induced by hydrogen peroxide (H_2_O_2_) exposure through the inhibition of proteasomal appoptosin degradation. Furthermore, we demonstrate that overexpression of appoptosin induces apoptosis through the JNK-FoxO1 pathway. Importantly, knockdown of appoptosin can ameliorate H_2_O_2_-induced JNK activation and apoptosis in primary neurons. Thus, we propose that appoptosin functions as an upstream regulator of the JNK-FoxO1 pathway, contributing to cell death in response to oxidative stress during neurodegeneration.

## Introduction

Reactive Oxidative Species (ROS) are primarily generated in mitochondria as natural by-products of oxidative respiration. ROS normally play an important role in cell signaling and homeostasis (Devasagayam et al., 2004). Transient fluctuations in ROS are counterbalanced by antioxidant mechanisms within the cell comprising non-enzymatic molecules and enzymatic scavengers (Birben et al., 2012). However, ROS production can also be induced in cells, or absorbed directly from the extracellular environment when cells encounter environmental insults (Martindale and Holbrook, 2002; Ma et al., 2017). In these instances, ROS may be deleterious to cell function and survival.

In humans, the brain represents ~2% total body weight but disproportionately consumes ~20% of total oxygen and caloric intake (Jain et al., 2010). Due to an elevated rate of aerobic metabolism and potential insufficiencies in antioxidant and scavenging enzymes, the brain predisposes to be particularly vulnerable to oxidative stress insults. Despite its heterogenous nature, oxidative stress is a common feature of neurodegenerative diseases such as Alzheimer's disease (AD), Parkinson's disease (PD), amyotrophic lateral sclerosis (ALS), and Huntington's disease (HD) (Ma et al., 2017; Cao et al., 2018). In addition, it has been proposed that several key proteins implicated in these neurodegenerative disorders (Aβ in AD, α-synuclein in PD, mSOD1 in ALS, frataxin in Friedreich's ataxia and α-B-crystallin in cataracts), may feature aberrant accumulation of Cu^2+^ and Fe^3+^ which catalyze the conversion of O_2_ to ROS, thereby exposing neurons to oxidative stress (Barnham et al., 2004; Zhou and Tan, 2017).

Appoptosin is a protein that resides in the mitochondrial inner membrane, and facilitates the exchange of glycine and 5-aminolevulinic acid between the cytosol and mitochondria during heme synthesis (Guernsey et al., 2009). Mutations in Appoptosin have been reported to be genetically linked to congenital sideroblastic anemia (Guernsey et al., 2009; Kannengiesser et al., 2011). We have previously demonstrated that appoptosin is a pro-apoptotic protein that can activate intrinsic caspase cascades, leading to neuronal apoptosis (Zhang et al., 2012). In addition, appoptosin levels are found to be increased in neurodegenerative diseases such as AD and progressive supranuclear palsy (PSP), and under oxidative conditions such as rodent stroke models and Aβ- or glutamate-treated neurons (Zhang et al., 2012; Zhao et al., 2015). However, it remains unclear how appoptosin is upregulated to cause cell death in neurodegeneration or under oxidative stress conditions. Here, we find that appoptosin levels are induced with exposure to H_2_O_2_, where appoptosin turnover is attenuated through proteasomal degradation with reactive oxygen stress. Moreover, we find that appoptosin mediates activation of ROS-induced JNK/FoxO1 pathways. Together, these results implicate appoptosin as an important mediator of ROS-induced signal transduction pathways and define pathways that regulate appoptosin expression. Given that appoptosin upregulation triggers pathogenic effects in AD and PSP, our results suggest that antioxidants may reverse neurodegeneration by mediating appoptosin turnover.

## Materials and Methods

### Cells, Vectors, Antibodies, and Reagents

HEK293T and SY5Y cells were maintained in high glucose DMEM with 10% FBS and penicillin/streptomycin. Primary cortical neurons from embryonic day 17 (E17) mouse embryos were maintained in neurobasal medium supplemented with B27 and 0.8 mM Glutamine.

The vector expressing myc-appoptosin was generated previously (Zhang et al., 2012). A FLAG-tagged control vector and vectors expressing FoxO1, FoxO1-AAA (T24A, S256A, and S319A) and FoxO1 short hairpin RNA (shRNA) were kindly provided by Dr. Jie Zhang (Institute of Neuroscience, Xiamen University). The HA-Ub vector was kindly provided by Dr. Hongrui Wang (School of Life Sciences, Xiamen University). Gene-specific shRNA sequences were designed using the Genelink website (www.genelink.com/sirna/shRNAi.asp) and annealed shRNAs were inserted into the pLL3.7 vector. Three different shRNA-expressing constructs were generated, and RNA downregulation was tested by real-time PCR and western-blot. Sequences targeting appoptosin in constructs showing RNAi activity were as follows: Human appoptosin 5′-GGATGTTGGCTGTACTCTT-3′ and 5′-ATTCAGAACTCACGTCCGT-3′; mouse appoptosin 5′-gtgatcaagacacgctatg-3′; Scrambled shRNA 5′-GCCATATGTTCGAGACTCT-3′.

The following antibodies were used in this study: anti-appoptosin (#ab133614) from Abcam; anti-Myc (9E10, sc-40) from Santa Cruz; anti-tubulin (MABT205) from Millipore; anti-cleaved PARP (#5625), anti-AKT (#4691), anti-β-catenin (#8480), anti-β-actin (#8457), anti-cleaved caspase-3 (#9661), anti-FoxO1 (#2880), anti-p-JNK (Thr183/Tyr185) (#9251), and anti-p-c-Jun (Ser73) (#3270) from Cell Signaling Technology. Horseradish peroxidase (HRP)-conjugated goat anti-rabbit IgG (H+L) secondary antibody (#31460) and HRP-conjugated goat anti-mouse IgG (H+L) secondary antibody (#31430) were from Thermo Fisher Scientific.

Hydrogen peroxide (H_2_O_2_), nicotinamide (NAM), and resveratrol were from Bio Basic Inc. Cycloheximide (CHX), MG132, SP600125, 4′,6-diamidino-2-phenylindole (DAPI) and 2′,7′-dichlorodihydrofluorescein diacetate (DCFH-DA) were from Sigma Aldrich. Protease and phosphatase inhibitor cocktails were from Roche.

### Vector Transfection

HEK293T cells were transfected using Turbofect (Thermo Fisher Scientific) according to the manufacturer's instructions. For transient co-expression of shRNAs and proteins, cells were firstly transfected with shRNA-expressing plasmids for 24 h, and subsequently with overexpression plasmids for an additional 24 h.

### Real-Time PCR

RNA was extracted using TRIzol Reagent (Invitrogen). Reverse transcription was performed using the ReverTra Ace qPCR RT Kit (Toyobo). Equal amounts of cDNA from each sample were subjected to real-time PCR experiments. Primers used in this study were as follows:

human appoptosin:

forward primer: 5′-GTCGGAGACACGGTGGAAAC-3′;
reverse primer: 5′-GCCAACATCCCAACACGTCTA-3′;

mouse appoptosin:

forward primer: 5′-GAAGGTGGTTCGCACAGAAAG-3′;
reverse primer: 5′-CCTCGCAAGAAATACTGCTTCG-3′;

18S:

forward primer: 5′-CGACGACCCATTCGAACGTCT-3′;
reverse primer: 5′-CTCTCCGGAATCGAACCCTGA-3′.

### Mitochondria Isolation

The cell mitochondria isolation kit was purchased from Beyotime. Mitochondria-cytosol fractionation was performed following manufacturer's instructions. Briefly, cells were pelleted by centrifugation at 600 g for 5 min at 4°C, re-suspended in mitochondrial isolation solution, and homogenized on ice using a tight-fitting pestle attached to a homogenizer. The homogenate was then centrifuged at different speeds to separate intact cells, mitochondria, and cytosol fractions.

### Adeno-Associated Virus (AAV) Packaging and Infection

AAV was packaged by Obio Technology (Shanghai) and supplied in liquid form (multiplicity of infection (MOI) equals to 1 × 10^12^). Primary cortical neurons were cultured and infected with AAV particles (MOI equals to 2 × 10^9^) on day 3 *in vitro* (DIV) and incubated for an additional 6 days before subsequent treatment.

### Western Blot

Cells were lysed in TNEN lysis buffer (50 mM Tris, pH 8.0, 150 mM NaCl, 2 mM EDTA, 1% Nonidet P-40), and supplemented with a protease inhibitor mixture. Equal amounts of protein lysates were resolved in SDS-polyacrylamide gel electrophoresis, transferred to polyvinylidene difluoride (PVDF) membranes, and probed with antibodies as indicated. Relative intensity of protein bands was quantified by densitometry with Image J.

### Cellular ROS Assay

Cellular ROS levels were evaluated by oxidation sensitive fluorescent probe DCFH-DA. Briefly, HEK293T cells were transfected with control or appoptosin plasmid for 24 h. After washing with PBS for three times, cells were incubated with cell culture media containing 10 μM DCFH-DA for 30 min. Cells were then washed with PBS for another three times. Fluorescence was observed under a fluorescence microscope; and fluorescence intensity was measured by Image J.

### Statistics

Statistical analysis was performed using GraphPad Prism 5 software. Results are presented as mean ± standard error of the mean (SEM). Unpaired *t*-test, one way or two-way ANOVA was used to assess statistical significance between groups.

## Results

### H_2_O_2_ Treatment Elevates Appoptosin Protein Levels

**Figure 1:**
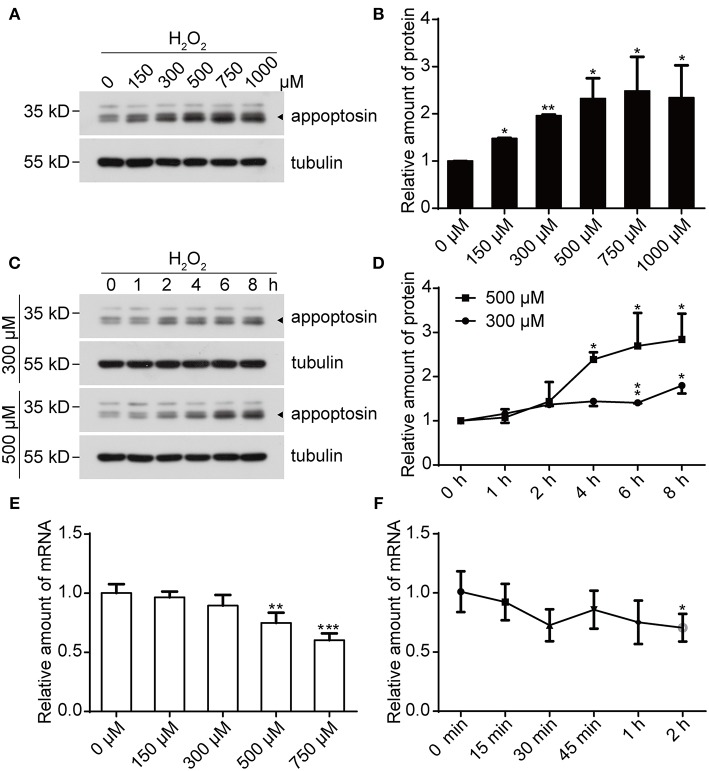
H_2_O_2_ treatment increases appoptosin protein levels. **(A)** HEK293T cells were exposed to varying H_2_O_2_ concentrations for 8 h. Appoptosin protein levels were analyzed by western-blot. **(B)** Quantification of results from **(A)**. **(C)** HEK293T cells were treated with 300 or 500 μM H_2_O_2_ for the time duration indicated. Appoptosin protein levels were determined by western-blot. **(D)** Quantification of results from **(C)**. **(E)** HEK293T cells were treated with increasing H_2_O_2_ concentrations for 8 h and appoptosin mRNA levels were determined by quantitative real-time PCR analysis. **(F)** Appoptosin mRNA levels in HEK293T cells treated with 300 μM of H_2_O_2_ for the time duration indicated as measured by quantitative real-time PCR. *n* ≥ 3; *P < 0.05, **P < 0.01, ***P < 0.001 (one-way ANOVA).

We previously reported that appoptosin is upregulated in primary cortical neurons after Aβ or glutamate exposure (Zhang et al., 2012). Because both Aβ (Behl et al., 1994) and glutamate (Parfenova et al., 2006) partially exert toxic effects through oxidative stress, it is likely that appoptosin expression is regulated through pathological oxidative insults. To test this, we treated cells with H_2_O_2_, a common membrane-permeable oxidant which is catalytically converted to reactive hydroxyl radicals (•OH) (Valko et al., 2005; Bienert et al., 2007). We found that exposure of HEK293T (Figures 1A,B) and SY5Y (Supplementary Figure 1) cells to H_2_O_2_ resulted in increased appoptosin levels in a dose- and time-dependent manner (Figures 1C,D). Interestingly, appoptosin mRNA levels were slightly reduced in a dose- (Figure 1E) and time-dependent (Figure 1F) manner upon H_2_O_2_ treatment. These results suggest that H_2_O_2_-dependent induction of appoptosin levels occurs through appoptosin-associated translation or turnover, rather than modulation of appoptosin mRNA transcripts.

### Appoptosin Is Degraded Through the Proteasomal Degradation Pathway

**Figure 2:**
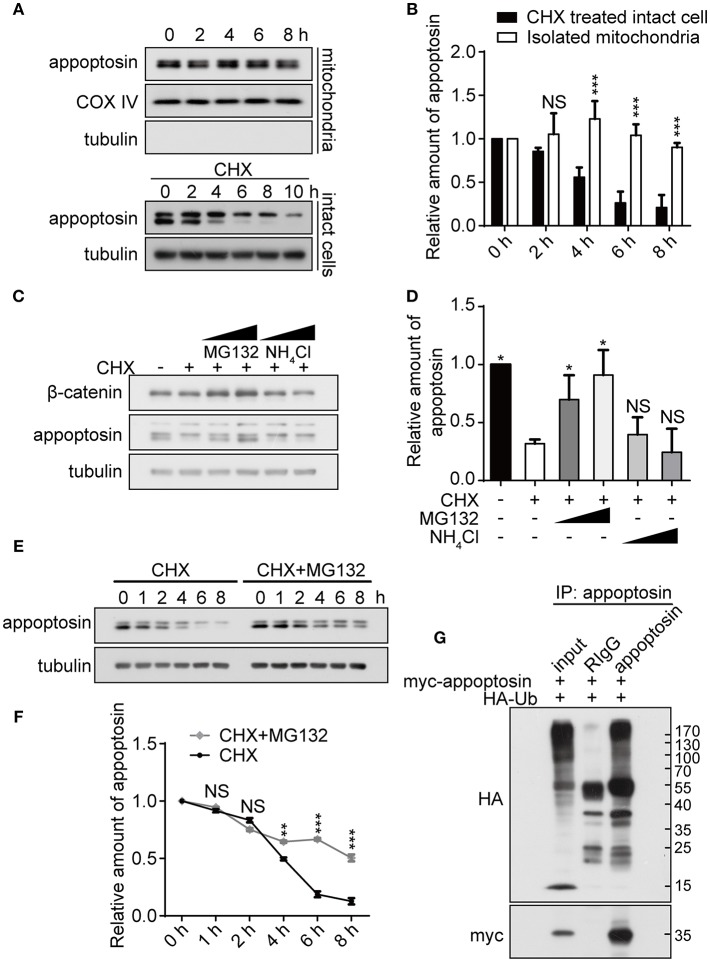
Appoptosin is degraded through the ubiquitin-proteasome pathway. **(A)** Appoptosin levels in isolated mitochondria or intact cells treated with cycloheximide (CHX) as determined by western-blot. **(B)** Quantification of results from **(A)**. **(C)** HEK293T cells were treated with DMSO alone (–), DMSO+CHX (50 μM), CHX+MG132 (10 and 20 μM), and CHX+NH_4_Cl (20 and 30 mM) for 8 h. β-catenin and appoptosin levels were determined by western-blot. **(D)** Quantification of results from **(C)**. **(E)** HEK293T cells were treated with CHX or CHX+MG132 (10 μM) for various time periods. Appoptosin levels were analyzed by western-blot. **(F)** Quantification of results from **(E)**. **(G)** HEK293T cells were co-transfected with HA-ubiquitin (Ub) and myc-appoptosin plasmids for 24 h. Equal protein amounts of cell lysates were subjected to immunoprecipitation (IP) with an appoptosin antibody or rabbit IgG, and then western-blot with HA and myc antibodies. Ten percent of lysates used for IP was immunoblotted in inputs. *n* ≥ 3; *P < 0.05, **P < 0.01, ***P < 0.001 (unpaired *t*-test or two-way ANOVA).

As previous studies have established that severe oxidative stress has an inhibitory effect on protein translation (Patel et al., 2002; Shenton et al., 2006; Ling and Soll, 2010), we characterized mechanisms underlying appoptosin degradation and stability. Appoptosin is an intra-mitochondrial transporter localized in the mitochondrial inner membrane. Mitochondrial proteins can be degraded by intrinsic mitochondrial proteases, or through lysosome or ubiquitin-proteasome systems (Ashrafi and Schwarz, 2013). To determine whether appoptosin is degraded through intrinsic mitochondrial pathways, we isolated mitochondria from HEK293T cells and assayed protein stability at 37°C at varying timepoints by immunoblot as described previously (Azzu et al., 2008). Minimal appoptosin turnover was observed in isolated mitochondria (Figures 2A,B). However, when intact HEK293T cells were incubated with cycloheximide (CHX) to inhibit protein synthesis, appoptosin was found to be rapidly degraded with a half-life of ~4 h (Figures 2A,B). These results suggest that appoptosin degradation is regulated by non-mitochondria associated pathways. Although the mitochondria inner membrane and lumen are spatially separated from ubiquitin-proteasomal machinery in the cytosol, cumulative evidence indicates that the ubiquitin-proteasome system is an important regulator for mitochondrial quality control. Proteins residing in the mitochondrial outer-membrane (Karbowski and Youle, 2011; Yoshii et al., 2011), inter-membrane space (Bragoszewski et al., 2013), and inner-membrane (Azzu and Brand, 2010) have been reported to be degraded through the ubiquitin-proteasome pathway. Here, we also pharmacologically inhibited proteasome and lysosome function with MG132 (10 or 20 μM) and NH_4_Cl (20 or 30 mM), respectively, in HEK293T (Figures 2C,D) and SY5Y (Supplementary Figure 2) cells. CHX-mediated inhibition of protein synthesis dramatically decreased appoptosin levels, which was dose-dependently reversed by MG132 but not NH_4_Cl. In addition, MG132 treatment significantly reduced appoptosin turnover kinetics (Figures 2E,F). These results indicate that proteasomal mechanisms also mediate appoptosin turnover and stability.

Since poly-ubiquitination is the obligatory initiating step during proteasome-mediated protein turnover (Murakami et al., 1992), immuno-precipitation was performed to determine whether appoptosin could be subjected to ubiquitination. Our results indicate that ubiquitin coprecipitated with appoptosin in HEK293T cells, confirming that appoptosin is indeed ubiquitinated (Figure 2G). Taken together, our results suggest that appoptosin is primarily degraded through the proteasome pathway.

### H_2_O_2_ Inhibits Appoptosin Turnover

**Figure 3:**
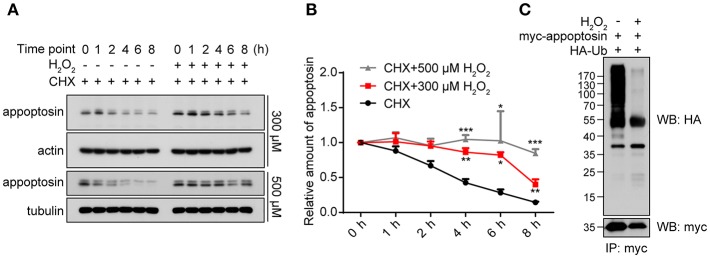
Appoptosin turnover is delayed under oxidative stress. **(A)** HEK293T cells were treated with cycloheximide (CHX) or CHX+H_2_O_2_ (300 or 500 μM) for the time indicated. Appoptosin levels were determined by western-blot. **(B)** Quantification of results from **(A)**. **(C)** HEK293T cells were co-transfected with HA-ubiquitin (Ub) and myc-appoptosin plasmids for 24 h. Cells were then treated with or without 300 μM H_2_O_2_ for 8 h. Equal protein amounts of cell lysates were subjected to immunoprecipitation (IP) with a myc antibody and then western blot with HA and myc antibodies. *n* ≥ 3; *P < 0.05, **P < 0.01, ***P < 0.001 (two-way ANOVA).

As a fundamental component that mediates cellular protein degradation (Davies, 2001; Jung and Grune, 2008), proteasomal function is potentially impaired during aging (Bulteau et al., 2000; Carrard et al., 2002) and under severe oxidative stress (Breusing and Grune, 2008; Wang et al., 2010). Moreover, activity of E3 ubiquitin ligases has been reported to be diminished under oxidative stress, leading to reduced ubiquitination and aberrant stabilization/accumulation of various protein species (Banerjee et al., 2010; Messina et al., 2012). We therefore tested whether H_2_O_2_ stabilizes appoptosin through the inhibition of proteasome-mediated degradation pathways. Our results indicate that appoptosin turnover kinetics were dose-dependently impaired by H_2_O_2_ (Figures 3A,B). In addition, H_2_O_2_ dramatically inhibited appoptosin ubiquitin conjugation (Figure 3C), indicating that the proteasomal degradation of appoptosin may be impaired under oxidative stress.

### Appoptosin Mediates the JNK Pathway Activation Under Oxidative Stress

**Figure 4:**
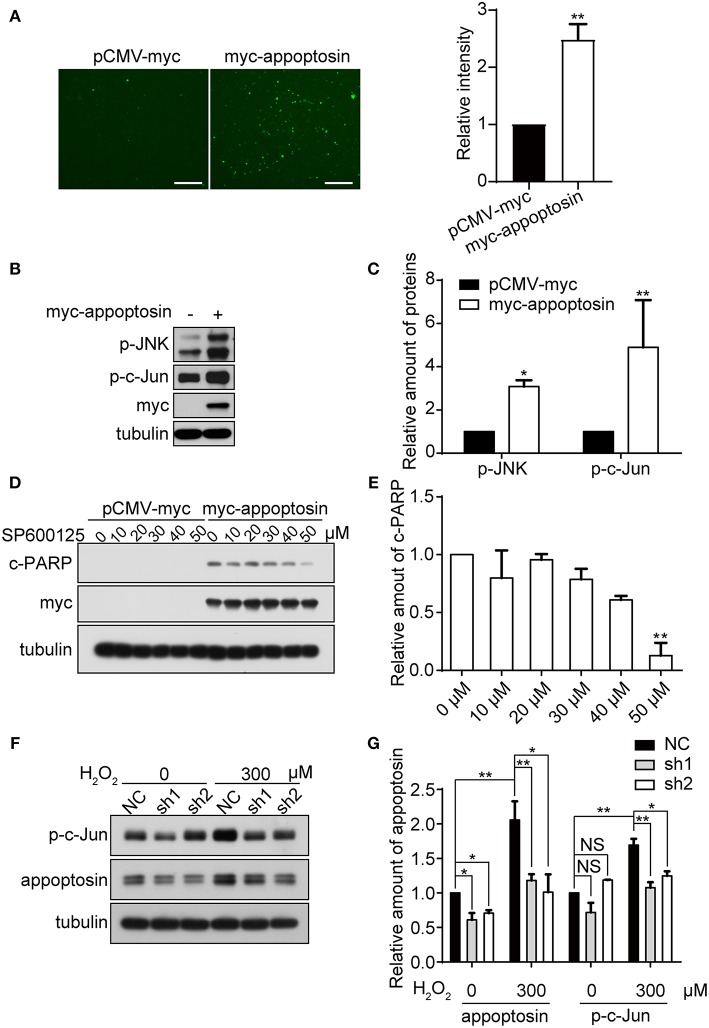
Appoptosin mediates induction of the JNK pathway with oxidative stress. **(A)** HEK293T cells were transfected with pCMV-myc or myc-appoptosin for 24 h. Cells were then washed and incubated with DCFH-DA (fluorescent probe as indicator of ROS) for 30 min. Fluorescence intensity was measured by Image J for comparison. Scale bar: 200 μm. **(B)** HEK293T cells were transfected with control or myc-appoptosin plasmids for 24 h. Cell lysates were analyzed for phosphorylated JNK (p-JNK) and phosphorylated c-Jun (p-c-Jun) by western-blot. **(C)** Quantification of results from **(B)**. **(D)** HEK293T cells were transfected with control pCMV-myc or myc-appoptosin plasmids for 5 h, and treated with DMSO or the JNK inhibitor SP600125 as indicated for additional 19 h. Cleaved PARP (c-PARP) and myc-appoptosin levels were analyzed by western-blot. **(E)** Quantification of results from **(D)**. **(F)** Cells were transfected with plasmids expressing scrambled shRNA (NC) or appoptosin shRNAs (sh1 and sh2) for 24 h, and treated with 0 or 300 μM H_2_O_2_. Levels of p-c-Jun and appoptosin were determined by western-blot. **(G)** Quantification of results from **(F)**. *n* ≥ 3; *P < 0.05, **P < 0.01 (one-way or two-way ANOVA).

Oxidative stress is tightly linked to downstream signaling cascades to regulate cell growth, senescence, and apoptosis (Martindale and Holbrook, 2002). We have previously shown that overexpression of appoptosin leads to aberrant overproduction of heme, and release of ROS (Zhang et al., 2012). Here we confirmed the effects of appoptosin overexpression on promoting ROS production, as evidenced by DCFH-DA staining (Figure 4A).

Although overexpression of appoptosin eventually activates intrinsic caspase-dependent apoptotic pathways (Zhang et al., 2012), the role of appoptosin in downstream ROS-responsive signaling pathways has not been elucidated. Mitogen-activated protein kinase (MAPK) pathways, including extracellular signal regulated kinases (ERK-1/2), c-Jun NH2-terminal kinases (JNK-1/2/3), and p38 MAPK proteins (p38 α/β/γ/δ), are related kinase cascades that connect numerous intra- and extra-cellular signaling pathways. JNK-MAPK proteins are strongly tied to stress response (Martindale and Holbrook, 2002), and numerous reports indicate that JNK-MAPK proteins are involved in the regulation of apoptosis during oxidative injury (Yin et al., 2000; Hreniuk et al., 2001; Kanayama and Miyamoto, 2007; Chen et al., 2008; Conde De La Rosa et al., 2008). Here, we observed that overexpression of appoptosin in HEK293T cells activated JNK and its downstream substrate c-Jun, as evidenced by upregulated p-JNK and p-c-Jun (Figures 4B,C). Pharmacological inhibition of JNK by SP600125 dose-dependently reduced appoptosin-induced apoptosis (Figures 4D,E). H_2_O_2_ treatment significantly increased p-c-Jun levels, which was fully reversed with shRNA-mediated appoptosin depletion (Figures 4F,G). These results suggest that appoptosin mediates H_2_O_2_-induced apoptosis through the JNK-MAPK pathway.

### FoxO1 Mediates Appoptosin-Induced Apoptosis

**Figure 5:**
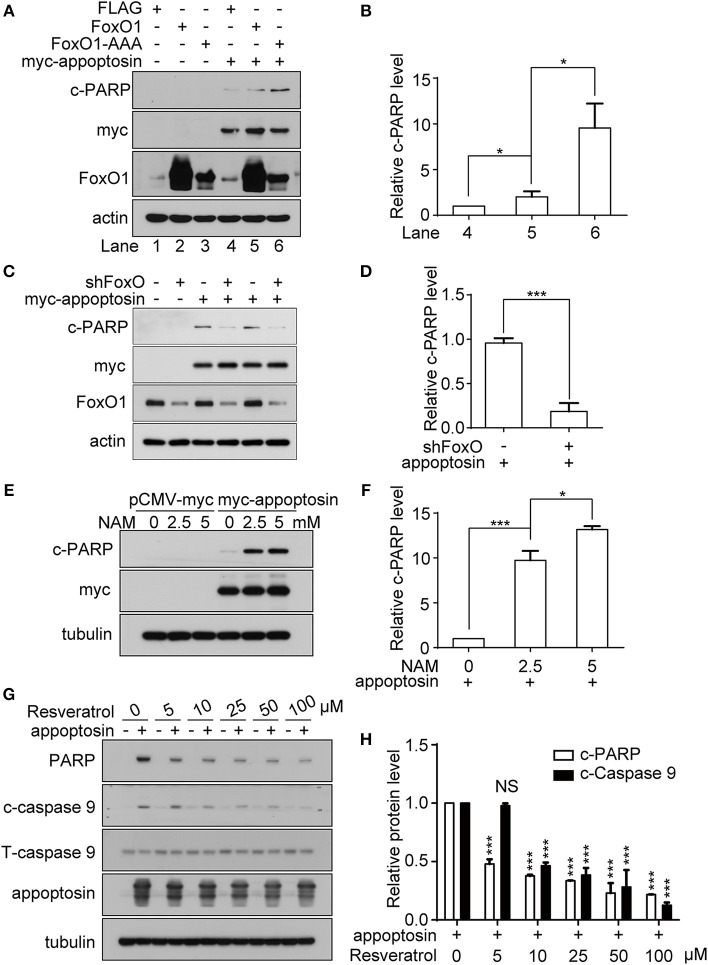
FoxO1 potentiates appoptosin-induced apoptosis. **(A)** HEK293T cells were transfected with plasmids expressing FLAG (control vector), FoxO1, or FoxO1-AAA plasmids for 24 h, and subsequently transfected with pCMV-myc or myc-appoptosin for another 24 h. Cleaved PARP (c-PARP), myc-appoptosin and FoxO1 levels were analyzed by western-blot. **(B)** Quantitative analysis of c-PARP levels in **(A)**. **(C)** HEK293T cells were transfected with scrambled control shRNA or FoxO1-shRNA (shFoxO) for 24 h, and subsequently transfected with pCMV-myc or myc-appoptosin for additional 24 h. Indicated protein levels were analyzed by western-blot. **(D)** Quantification of results from c-PARP levels in **(C)**. **(E)** HEK293T cells were transfected with pCMV-myc or myc-appoptosin for 6 h, and subsequently treated with the SIRT1 inhibitor NAM for 18 h as indicated. Levels of c-PARP and myc-appoptosin were determined by western-blot. **(F)** Quantification of results from c-PARP levels in **(E)**. **(G)** HEK293T cells were transfected with pCMV-myc or myc-appoptosin for 6 h, and subsequently treated with the SIRT1 activator resveratrol for 18 h as indicated. Levels of c-PARP, cleaved caspase 9 (c-caspase 9), total caspase 9 (T-caspase 9) and appoptosin were analyzed by western-blot. **(H)** Quantification of results from c-PARP and c-caspase 9 levels in **(G)**. *n* ≥ 3; *P < 0.05, ***P < 0.001 (one-way ANOVA).

It has been previously established that the class O of forkhead box transcription factor, FoxO1 can trigger apoptosis in response to oxidative stress through the expression of apoptotic proteins such as FasL, Puma, TRAIL, and Bim (Cui et al., 2009). Studies have also shown that JNK can act as an upstream activator of FoxO-related transcription factors multiple levels (Van Der Horst and Burgering, 2007; Karpac and Jasper, 2009), to modulate cell metabolism, cell cycle inhibition, oxidative stress resistance, and/or apoptosis. Although we observe that overexpression of FoxO1 or its active form FoxO1-AAA (mutations at the AKT phosphorylation sites, which lead to nucleus retention) alone had no effect on apoptosis, co-expression of appoptosin together with FoxO1-AAA significantly potentiated appoptosin-induced apoptosis (Figures 5A,B). In contrast, downregulating FoxO1 expression by shRNA significantly inhibited appoptosin-induced apoptosis (Figures 5C,D).

Various post-translational modifications have been observed in FoxO1, including phosphorylation, acetylation, ubiquitination, and methylation (Calnan and Brunet, 2008). Among these, FoxO1 acetylation enhances the expression of genes involved in apoptotic pathways (Yang et al., 2009), whereas FoxO1 deacetylation promotes transcription of genes involved in DNA repair and stress resistance. Nicotinamide (NAM) is a well-known inhibitor of SIRT1 (Avalos et al., 2005), which modulates FoxO1 activity through deacetylation (Hariharan et al., 2010). We found that although NAM treatment had no effect on apoptosis in normal cells, NAM potentiated apoptosis in cells overexpressing appoptosin in a dose-dependent manner (Figures 5E,F). Conversely, resveratrol, a well-documented SIRT1 activator (Borra et al., 2005), dose-dependently inhibited appoptosin-induced apoptosis (Figures 5G,H). Together, these results indicate that the downstream JNK pathway effector, FoxO1, can potentiate appoptosin-associated apoptosis pathways.

### Downregulation of Appoptosin Protects Primary Neurons From Oxidative Injury

**Figure 6:**
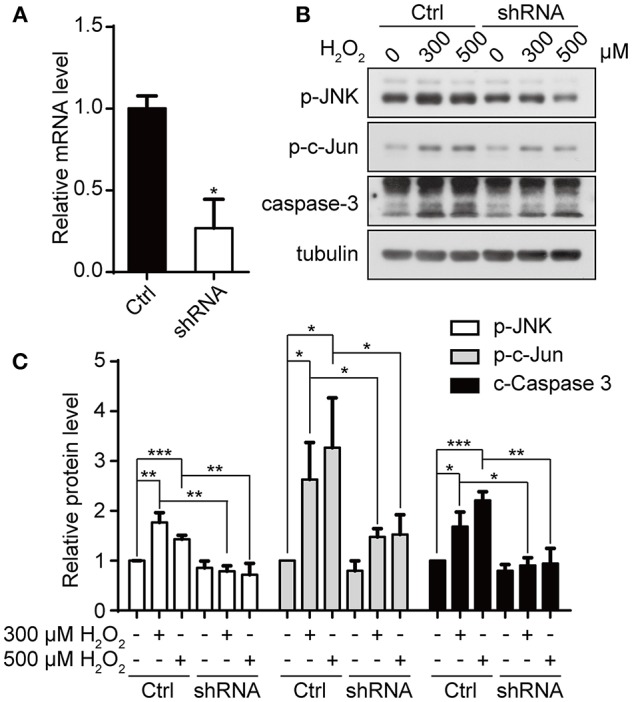
Downregulation of appoptosin protects primary neurons from oxidative injury. **(A)** Mouse primary neurons were infected with AAVs carrying scrambled control shRNA (Ctrl-AAV) or AAVs carrying an shRNA targeting appoptosin (shRNA-AAV) for 72 h. Appoptosin mRNA levels in neurons infected with Ctrl-AAV or shRNA-AAV were determined by quantitative real-time PCR. **(B)** Neurons infected with Ctrl-AAV or shRNA-AAV were treated with H_2_O_2_ (0, 300, or 500 μM) for 4 h. Levels of p-JNK, p-c-Jun and caspase 3 were analyzed by western-blot. **(C)** Quantification of results from **(B)**. *n* ≥ 3; *P < 0.05, **P < 0.01, ***P < 0.001 (one-way ANOVA).

We have previously shown that downregulation of appoptosin can protect neurons from Aβ and glutamatergic toxicity (Zhang et al., 2012). To investigate the effect of appoptosin downregulation on neuronal injury induced by oxidative stress, we transduced primary neurons with AAV expressing appoptosin-shRNA or control AAV on DIV-3 for 6 days, and subsequently exposed neurons to H_2_O_2_ for 4 h. Appoptosin mRNA levels in appoptosin shRNA-expressing cells were reduced to ~25% of the control (Figure 6A). Consistent with results from HEK293T cells, H_2_O_2_-induced JNK/c-Jun activation and caspase-3 cleavage were reversed by appoptosin downregulation (Figures 6B,C). These results indicate that reducing appoptosin expression can protect neurons from oxidative injury.

## Discussion

ROS relay signals as important second messengers, and serve important regulatory functions in cell growth and differentiation at very low concentrations (Suzuki et al., 1997; Sauer et al., 2001). However, excessive ROS-induced oxidative stress impairs vital cell components, leading to cell cycle arrest and eventual apoptosis or necrosis. These degenerative events are important contributors to multiple diseases and are particularly important in a spectrum of neurodegenerative disorders.

Mitochondria are the primary source for ROS generation (Circu and Aw, 2010), and are therefore immediately susceptible to ROS-mediated oxidative damage. With increasing ROS levels, organellar damage within mitochondria includes oxidative damage of mitochondrial DNA, lipids, and proteins, which can have deleterious effects on cell functions (Fariss et al., 2005; Gibson, 2005; Circu et al., 2009; Rachek et al., 2009; Andreazza et al., 2010). Additionally, mitochondria play a central role in mediating intrinsic apoptotic pathways. As a component of the mitochondrial inner membrane, appoptosin and its overexpression have recently been shown to induce apoptosis by increasing heme synthesis, ROS production and cytochrome c release. Appoptosin expression is found to be pathologically upregulated in AD and PSP disorders that are also associated with oxidative stress. In the present study, we found that appoptosin protein levels accumulated under oxidative stress through impaired proteasome-mediated appoptosin turnover. H_2_O_2_-induced effects on protein degradation appear to be specific to appoptosin, since H_2_O_2_ treatment had no effect on other proteasomal substrates such as SIRT1 (Caito et al., 2010) and FoxO1 (Huang et al., 2005). In addition, we found that appoptosin could mediate the JNK activation induced by oxidative stress, and downregulation of appoptosin attenuated H_2_O_2_-induced apoptosis. JNK is activated by the MAPKKK (ASK1) during oxidative stress and is believed to be a central regulator of both intrinsic and extrinsic apoptotic pathways (Sinha et al., 2013). JNK directly or indirectly activates apoptotic pathways by phosphorylating effector proteins (such as Bim, Bad, and Bmf) or transcription factors [such as c-Jun, FoxOs, p53, and c-myc (Sinha et al., 2013)]. Given its role in ROS production shown previously (Chambers and Lograsso, 2011), it is likely that appoptosin activates JNK through ROS induction and mitochondrial fission (Zhang et al., 2016).

Here we observe that overexpression of the transcription factor FoxO1 potentiates appoptosin-induced apoptosis. Downregulation of FoxO1 likewise attenuated appoptosin-induced apoptosis, and the SIRT1 inhibitor NAM promoted appoptosin-induced apoptosis, whereas treatment with the SIRT1 activator resveratrol blocked appoptosin-induced apoptosis. As SIRT1 modulates FoxO1-dependent transcriptional activity to promote expression of genes involved in stress resistance while inhibiting genes that trigger apoptosis, our results indicate that FoxO1 functions downstream of appoptosin. These results produce a working model where FoxO1 functions downstream of JNK, whereby FoxO1 triggers apoptosis under oxidative stress.

## Data Availability

The data for this manuscript will be available from the authors to qualified researchers upon reasonable request. Requests for access to the data should be directed to the corresponding authors.

## Ethics Statement

All animal procedures were approved by the Laboratory Animal Management and Ethics Committee of Xiamen University.

## Author Contributions

CZ, ZT, and YX performed the experiments. CZ, YZ, and XZ interpreted experimental data. YZ, TH, and HX reviewed the manuscript. ZL, HL, and DC edited the manuscript. CZ, Y-wZ, and XZ wrote the manuscript. All authors read and approved the final manuscript.

### Conflict of Interest Statement

The authors declare that the research was conducted in the absence of any commercial or financial relationships that could be construed as a potential conflict of interest.
