# Supplementary material for: Appoptosin Mediates Lesions Induced by Oxidative Stress Through the JNK-FoxO1 Pathway
Source: Front Aging Neurosci. 2019 Sep 4;11:243. doi: 10.3389/fnagi.2019.00243 (PMC6737070; doi:10.3389/fnagi.2019.00243)
Supplement: Supplementary file 1 [file Data_Sheet_1.PDF]

Supplementary Material

1 Supplementary Figures

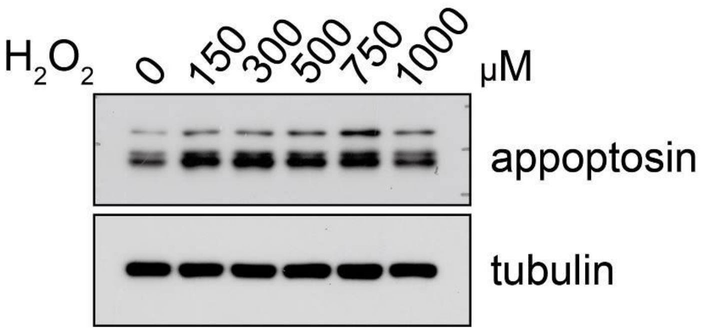

Supplementary Figure 1. H_2O_2 dose-dependently up-regulates appoptosin levels. SY5Y cells were treated with H_2O_2 for 8 h as indicated. Cell lysates were subjected to western-blot analysis.

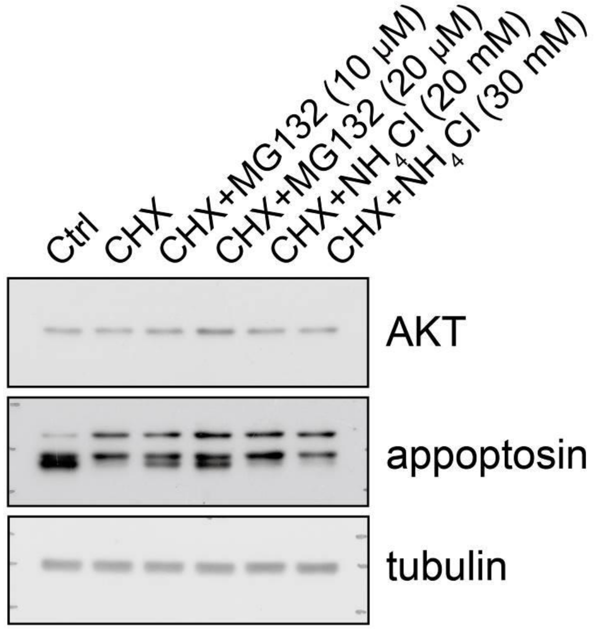

Supplementary Figure 2. Appoptosin degradation is inhibited by the proteasome inhibitor MG132. SY5Y cells were treated with DMSO (as control, Ctrl), cycloheximide (CHX, 50 µM), CHX+MG132 (10 and 20 µM), and CHX+NH_4Cl (20 and 30 mM) for 8 h. Cell lysates were subjected to western-blot analysis for appoptosin and AKT.
